# Factors Associated with Marital Satisfaction and Quality of Life in Family Caregivers of Patients with Mental Disorders

**DOI:** 10.3390/ijerph16162825

**Published:** 2019-08-08

**Authors:** Douglas José Nogueira, Ruth Minamisava, Sheila Araujo Teles, Sandra Maria Brunini de Souza, Jacqueline Andréia Bernardes Leão Cordeiro, Denise Soares de Cirqueira, Virginia Visconde Brasil, Ana Lúcia Rezende Souza, Karlla Antonieta Amorim Caetano, Lizete Malagoni de Almeida Cavalcante Oliveira, Diane Maria Scherer Kuhn Lago, Maria Alves Barbosa

**Affiliations:** 1 Faculty of Nursing, Federal University of Goias, Goiânia 74605-080, Brazil; 2 Secretariat of Health, Goiânia 74884-900, Brazil; 3 Faculty of Physical Therapy, Federal University of Jataí, Jataí 75804-020, Brazil; 4 Faculty of Ceilandia, Brasilia University, Brasilia 72220-275, Brazil

**Keywords:** quality of life, caregivers, mental disorders, marital satisfaction, crisis

## Abstract

(1) Background: The aim of this research was to analyze factors associated with quality of life (QoL) and marital satisfaction in married family caregivers of patients with mental disorders. (2) Methods: A cross-sectional study was conducted in all community mental health services in Goiania municipality, Brazil, in 2016–2017. Married family caregivers of patients with severe and persistent mental disorders were recruited and their QoL and marital satisfaction was assessed by using the World Health Organization Quality of Life Instrument Abbreviated version (WHOQOL-BREF) and Marital Satisfaction Scale. Multiple linear regressions were performed to identify factors associated with QoL and marital satisfaction. (3) Results: For 163 family caregivers, the psychological and environmental QoL domains presented the best and the worst scores, respectively. Factors independently associated with better QoL for caregivers were male caregiver, the younger age of a caregiver, >8 years of schooling, ≥5 years as a caregiver who performed physical activities, caregiver without chronic disease, and no patient’s crisis in the last 30 days. Factors independently associated with marital satisfaction of the caregiver were male caregiver, caregiver with >8 years of schooling, caregiver who received support by relatives to care for the patient, caregiver who performed physical activities, no patient’s crisis in the last 30 days, and patient hospitalization in the last six months; (4) Conclusions: The main predictor for marital satisfaction was support by relatives, and for QoL it was no patient’s crisis in the last 30 days.

## 1. Introduction

Approximately one in five individuals of the population are affected by a common mental disorder in the last 12 months of their life [1]. The closure of psychiatric hospitals in recent decades resulted in an increase of patients with mental disorders being treated in the community mental health services. The deinstitutionalization of these patients was a benefit, but it raised the responsibility of families for caring for psychiatric patients since the caregiving is mostly shared among close relatives [2].

The relatives of patients with mental disorders have assumed the key role of primary caregivers in both periods of relative stability and during relapses or crisis, when the patients suffer exacerbations of acute symptoms. An informal caregiver is an unpaid individual (i.e., partner, family member, friend, or neighbor) involved in assisting others with activities of daily living and/or medical tasks.

The current literature on caregivers of patients with mental disorders is dominated by studies on family caregivers. In fact, family caregivers can have an additional suffering by watching someone they love suffer. The effect of caring for a patient with a mental disorder on the quality of life (QoL) of family caregivers has been highlighted in previous reports [3,4,5,6]. The low QoL of caregivers, probably, comes from reactions to the illness, changes in the household routine, and/or a reduction in leisure activities [7]. Furthermore, family caregivers can experience problems in intra-family relationships, undergo economic hardships, suffer from the effects of social stigma, and are more likely to have family burdens [8]. Some factors are related to the QoL of the family caregiver, including the duration of the mental disorder [9], the number of psychiatric hospitalizations [10], educational level [11], support for caring [12], and positive and negative symptoms [9]. However, surprisingly little is known about the QoL of married family caregivers.

Likewise, few studies have been conducted on marital satisfaction among married caregivers. In the UK, young patients with Parkinson’s disease had worse scores on the marital satisfaction (marital harmony) scale [13]. Other investigations showed that fertile couples obtained significantly lower sexual satisfaction and marital satisfaction scores compared to the infertile ones [14]. A study with a spousal caregiver found a lower relationship satisfaction when care recipients were more depressed [15]. The sexual satisfaction among the spouses of mental disorder patients also decreased significantly over time [16]. It is possible that the married caregivers may feel forced to give up their leisure time, have to experience severe burdens, and may need support. Moreover, a study found that the odds of dying for married individuals who described their marriage as very happy were significantly lower than the odds of dying for married individuals who described their marriage as not too happy [17].

We hypothesized that the distress of the married family caregivers during the patient’s crisis may lead to a decrease of the quality of life and/or the marital satisfaction of these caregivers. Nevertheless, investigations carried out in Brazil have focused on the burden of married family caregivers of patients with mental disorders, leaving a gap in the field of marital satisfaction and QoL [18,19,20]. It is also unclear which factors could improve QoL and marital satisfaction for them. The objective of this study was to analyze the factors related to QoL and marital satisfaction in family caregivers of patients with mental disorders.

## 2. Materials and Methods

### 2.1. Study Design and Setting

A cross-sectional study was conducted in the municipality of Goiania, mid-western region of Brazil. Brazil is an upper-middle-income country; however, social inequality is still high. Approximately 70% of Brazilians use the Unified Health System (SUS), which offers healthcare free of charge, including psychiatric services. In 2017, Goiania had a population of an estimated estimated 1,466,105 inhabitants [21] and had four SUS community mental health services, which attended to adults with severe and persistent psychiatric diseases.

### 2.2. Participants

All family caregivers of patients with mental disorders attended to at the SUS community mental health services in Goiania municipality were eligible to participate in the study if the caregiver was married (or living as married) and aged 18 years or older. We considered the first-degree relatives or husband/wife who was involved in medical decisions, provided care, and carried out most treatment recommendations for patients with mental disorders as family caregivers. Married family caregivers who were in divorce processes and caregivers with severe mental disorders were excluded.

All subjects gave their informed consent for inclusion before they participated in the study. The study was conducted in accordance with the Declaration of Helsinki, and the protocol was approved by the Ethics Committee of the Clinicas Hospital of the Federal University of Goias (protocol #1,779,240).

### 2.3. Data Collection and Instruments

All family caregivers of patients with a mental illness were identified from medical records of all patients who attended the SUS community mental health services in Goiania municipality from February to August 2016. We made up to three attempts to make a phone call with each caregiver. When the eligibility criteria were met, the caregiver was invited to participate in the study. Face-to-face interviews were scheduled between September 2016 and March 2017 with family caregivers at the health service’s facilities or at the caregiver’s home. The interviewers were nurses and nursing students specially trained for the purpose of this study. This training included recommendations for interviewing, administering the QoL and marital satisfaction instruments, and principles of research ethics.

Data on caregiver and on patient were included in questionnaires. To analyze if the crisis of a patient with a mental disease can be related to the quality of life and marital satisfaction, potentially confounding variables were chosen based on biological plausibility. Variables on the married family caregivers were age in years, gender, years of schooling (≤8; >8 years), time as a caregiver (<5; ≥5 years), support by relatives to care for the patient (yes; no). The variables on the patient with a mental disorder obtained from medical records were gender, age, crisis in the last 30 days (yes; no), and international classification of the mental disorder. For the purpose of this study, the patient crisis was defined as any action, feelings, and behaviors of the patient which can lead to hurting themselves or others, and/or set them at risk of being unable to care for themselves or function in the community in a healthy manner.

The QoL was assessed using the World Health Organization Quality of Life Scale Abbreviated version (WHOQOL-BREF), translated and validated into Brazilian Portuguese [22]. The WHOQOL-BREF is a generic instrument of QoL assessment, self-administered, with 26 questions. The first question asks about an individual’s overall perception of quality of life and the second one asks about an individual’s overall perception of their health. The remaining 24 questions are subdivided into four domains: physical health (7 questions), psychological (6 questions), social relationships (3 questions), and environment (8 questions). Each question was rated from 1 to 5 (1 for very dissatisfied/very poor to 5 for very satisfied/very good).

The marital satisfaction was assessed by using the Marital Satisfaction Scale (MSS), developed in Mexico [23], translated and validated into Brazilian Portuguese [24]. The MSS considers marital satisfaction as the attitude towards the partner and the marital interaction. It is a self-applicable questionnaire with 24 items, all of them with three answer options: “I like it as it is”, “I wish it improves” and “It needs to change”. It gathers three sub-scales or aspects of marital satisfaction:

- Satisfaction with marital interaction with 10 items: The time my wife (husband) dedicates to our marriage; How often my wife (husband) says nice words to me; How much my wife (husband) attends me; How often my wife (husband) hugs me; The care my wife (husband) has for my appearance; The communication with my wife (husband); The behavior of my wife (husband) with other people; The way she (he) asks me to have sex; The time she (he) dedicates to me; The interest my wife (husband) has for what I do;
- Satisfaction with partner’s emotional aspects with 5 items: The way she (he) behaves when sad; The way she (he) behaves when upset; The way she (he) behaves when worried; The way she (he) behaves when in a bad mood; The reaction of my wife (husband) when I do not want to have sex; and
- Satisfaction with the way of organizing, rules establishment, and compliance by the partner with 9 items: The time she (he) spends for herself (himself); The way my wife (husband) organizes her (his) life and her (his) things; My wife’s (husband’s) priorities in life; The way she (he) spends her (his) free time; The punctuality of my wife (husband); My wife’s (husband’s) care for her (his) health; The time we spend together; The way my wife (husband) tries to solve problems; The rules my wife (husband) imposes in our home.

### 2.4. Statistical Analysis

A descriptive analysis of the predictor variables was performed to describe the results as numbers and proportions for categorial variables and as means with their respective standard deviation (sd) and median, as well as interquartile range and minimum and maximum values, for continuous variables (caregiver’s age, patient’ age). We calculated the proportions of diagnosis of mental disorders according to the International Classification of Diseases and Related Health Problems 10th revision. The chi-square or the Fisher’s test were used to detect differences in proportions of social-demographic characteristics among family caregivers who were and who were not married to a patient with a mental disorder.

Questions from WHOQOL-BREF were processed according to the recommendations of the WHOQOL group [25]. The scores of questions 3, 4, and 26 (1 = 5, 2 = 4, 3 = 3, 4 = 2, and 5 = 1) were recoded because they are negative questions. The mean of each domain was calculated of values varying from 0 to 100, which were multiplied by 4, minus 4, and multiplied by 100/16. Higher scores represent a better QoL. Questions from the MSS are scored from 1 to 3, and a mean was calculated for each sub-scale. Higher scores represent lower marital satisfaction.

We calculated means, standard deviations, 95% confidence intervals (95% CI), and minimum and maximum scores of QoL domains and MSS subscales. The means of scores were considered statistically different when the respective 95% CI did not overlap. The Cronbach’s Alpha was used to measure internal consistence for each WHOQOL-BREF domain and MSS subscale.

A multiple linear regression was conducted to identify the association between explanatory variables and each domain of QoL and each subscale for the marital satisfaction. Each explanatory variable was included in the final model based on biological plausibility and changes in the estimated exposure effect. All exposed variables were tested for collinearity through the tolerance and variance inflation factor. Regression coefficients (B), 95% CI, and *p*-values are presented as the final adjusted analysis. In all analyses, values were considered significant when *p* < 0.05. The data were analyzed using the SPSS software version 18.0.

## 3. Results

Of the 732 patients who attended the SUS community mental health services in Goiania municipality, 67 patients could not be reached, 152 patients had no caregiver, 235 caregivers were non-family caregivers, 98 caregivers were not-married family caregivers, 5 caregivers presented severe mental disorders, and 12 caregivers declined study participation. Overall, 163 married family caregivers were enrolled in the study. The mean age of the caregivers was 53 years (standard deviation = 12 years), the median age was 53 years (minimum = 22, maximum = 78 years), 93 (57.1%) were female, the mean value of years as a caregiver was 11.2 years (standard deviation = 11.6 years,) and median of years as a caregiver was 7 (minimum = 1, maximum = 50 years). Table 1 shows the description of the predictor variables for quality of life and for marital satisfaction of married family caregivers of patients with mental disorders.

Among the patients with mental disorders, 95 (58.3%) were female, the mean age was 46 years (standard deviation = 14 years), and the median age was 48 years (minimum = 19 and maximum = 75 years). These patients with mental disorders who were cared for by family members were diagnosed with schizophrenia, schizotypal disorders, and delusional disorders (*n* = 77; 47.2%); mood disorders (*n* = 52; 31.9%); neurotic disorders, stress related disorders, and somatoform disorders (*n* = 22; 13.5%); other mental disorders due to brain injury, dysfunction, and physical disease (*n* = 11; 6.75%); and organic mental disorder, including symptomatic ones (*n* = 1; 0.6%).

Family caregivers married to a patient with a mental disorder presented no significant differences in gender and age when compared with family caregivers married to someone other than the patient (*p* > 0.05).

For their overall perception of QoL, 1.8% of the caregivers reported very poor QoL, 19.0% poor QoL, 24.5% neither poor nor good QoL, 36.8% good QoL, and 17.8% very good QoL. Concerning the caregivers’ satisfaction with their own health, 35.0% were dissatisfied, 19.6% were neither satisfied nor dissatisfied, 31.90% were satisfied, and 13.5% were very satisfied.

The family caregivers of patients with mental disorders reported better scores for the psychological domain of QoL and worse scores for the environment domain of QoL (Table 2). The internal consistency of QoL domains ranged from acceptable (>0.70) to good (0.80 ≤ α < 0.90). Regarding marital satisfaction, the subscale ‘satisfaction with emotional aspects’ presented higher scores, that is, lower satisfaction with the partner’s emotional aspects. The items of the subscale ‘satisfaction with emotional aspects’ to which only ~25% of the caregivers answered “I like it as it is” were: the way she (he) behaves when sad; the way she (he) behaves when upset; and the way she (he) behaves when in a bad mood.

Table 3 shows that predictors associated with the best QoL in the physical domain were: male gender, ≥5 years as a caregiver, and absence of patient’s crisis in the last 30 days. Predictors associated with a better QoL in the psychological domain were: male gender, ≥5 years as a caregiver, and absence of patient’s crisis in the last 30 days. No predictors were found for QoL in the social relationships domain. Factors associated with a better QoL in the environment domain for caregivers were the caregiver with >8 years of schooling, ≥5 years as a caregiver, and absence of patient’s crisis in the last 30 days.

Table 4 shows the predictors related to the MSS subscales. Predictors of satisfaction with marital interaction were male gender, >8 years of schooling, and support by relatives. Predictors of satisfaction with emotional aspects were male gender, support by relatives, and absence of patient’s crisis in the last 30 days. Predictors of satisfaction with structural aspects were male gender and support by relatives.

## 4. Discussion

This study explored marital satisfaction and QoL in family caregivers of patients with mental disorder and predictors associated with them in Goiania municipality, Brazil. Overall, 20.86% of the caregivers reported a poor or very poor overall perception of QoL, and 35.0% were dissatisfied with their own health. The best score among QoL domains was the psychological domain and the worst, the environmental domain. Satisfaction with the partner’s emotional aspects obtained the worst rating among the MSS subscales. Caregivers of patients with mental disorders who presented a crisis in the last 30 days had a worse quality of life and lower marital satisfaction when compared to caregivers whose patients did not present a crisis in the last 30 days. On the other hand, the support of family members to care for the patient was not a predictor of better quality of life but was a predictor of better marital satisfaction.

Unlike our results, in Malaysia and Hong Kong, better QoL were identified in the physical health domain and lower in the psychological one [11,26]. In India, caregivers of patients with schizophrenia obtained higher score in the environment domain but lower in the social [27]. In the study with caregivers of patients with schizophrenia performed in Australia, the higher score was obtained in the physical health domain and the lower in the social relationships [28]. However, differences in social support, culture, health care systems, therapeutic goals of clinicians, and the daily lives of caregivers could allow for variation in the quality of life among caregivers of people with mental diseases. In the present study, the highest facet scores of the psychological domain were those related to low negative feelings, high spirituality/religion/personal beliefs, high self-esteem, and high bodily image and appearance. In fact, Brazilians are very optimistic people [29], which supports the view that optimism is associated with cultural dimensions [30]. Despite this, our results showed lower scores in the environmental domain, specifically in the facets of financial resources and participation in and opportunities for recreational/leisure activities. In fact, social inequalities in Brazil fluctuate over time but have remained high for decades [31], and most individuals who are SUS users belong to the lowest socioeconomic classes. Thus, caution should be used in comparing results from the current study with previous investigations.

Patients with a mental health crisis in the last 30 days were associated with both worse QoL and marital satisfaction of their caregivers. Health professionals may have difficulties in understanding the family’s experience in caring, which may limit the support to the family caregiver, since the suffering of the family caregiver may not be obvious to mental health professionals [32]. Symptoms of a mental health crisis may also disrupt the daily life of a family, becoming a source of concern and motivating to seek help from health services [33]. The psychoeducational intervention for family caregivers of individuals with mental disorders is presumably one of the ways to prevent a mental health crisis. However, that intervention seems to prevent relapse but there is no evidence of an improvement of the quality of life in family caregivers [34]. However, we should consider the delivery of technical support to the family caregivers in order to help them to better cope with the patient’s crisis in order to expand the caregiver’s autonomy. In addition to autonomy, caregivers who receive enough professional support from the health system may be able to reduce family conflicts or even marital satisfaction. Interestingly, the support by relatives predicts better marital satisfaction but not a better quality of life for married family caregivers.

This study presents some limitations. First, a selection bias might have been introduced by not including family caregivers of a person who was temporarily hospitalized and caregivers of patients with less severe diseases who were not attended to at the SUS community mental health services. The selection bias due to not including caregivers of patients with moderate and mild cases probably diminished both QoL and marital satisfaction scores. Second, the diagnosis of the mental health condition was based on patient records. Third, a recall bias was related to history of mental health crisis. Fourth, the cross-sectional design nature of the current research does not allow for establishing causal inferences. Finally, this study was conducted among family caregivers from a Brazilian city; thus, broad generalization should be avoided.

## 5. Conclusions

In the mid-western region of Brazil, the main factor associated with marital satisfaction in family caregivers of patients with mental disorders was to receive support by relatives in caring for the patient, while a patient with a mental health crisis in the last 30 days was the main factor associated with QoL. Further research should focus on expanding access to appropriate care to support married family caregivers.

## Figures and Tables

**Table 1:** Description of the predictor variables for quality of life and for marital satisfaction.

VARIABLES	*n*	%
Related to caregivers		
Gender		
Female	93	57.1
Male	70	42.9
Age group		
<60 years	115	70.6
≥60 years	48	29.4
Mean age in years (standard deviation)	53	(12)
Median age in years (interquartile range)	53	(45–62)
≤8 years of schooling		
no	58	35.6
yes	105	64.4
Years as caregiver		
<5	62	38.0
≥5	101	62.0
Support by relatives		
no	68	41.7
yes	95	58.3
Related to patient		
Gender		
Female	68	41.7
Male	95	58.3
Age group		
<60 years	135	82.8
≥60 years	28	17.2
Mean age in years (standard deviation)	46	(14)
Median age in years (interquartile range)	48	(36–57)
Crisis in the last 30 days		
no	123	75.5
yes	40	24.5

**Table 2:** Means, standard deviations (sd), minimum and maximum values, and Cronbach’s Alpha of scores of WHOQOL-BREF^a^ domains and of ESC^b^ subscales of family caregivers of patients with mental disorders.

DOMAIN SUBSCALES	Mean	SD	95% CI^c^	Range	Cronbach’s Alpha
WHOQOL-BREF ^a^					
***General questions***					
How would you rate your quality of life?	62.42	26.26	58.36–66.49	0.00–100.00	
How satisfied are you with your health?	55.98	26.91	51.82–60.14	25.00–100.00	
Domain					
Physical Health Domain	62.86	16.44	60.32–65.40	25.00–96.43	0.740
Psychological Domain	70.45	13.08	68.43–72.47	37.50–95.83	0.726
Social Relationships Domain	64.42	17.74	61.67–67.16	8.33–100.00	0.732
Environment Domain	50.38	18.86	47.47–53.30	6.25–87.50	0.831
MSS ^b^ SUBSCALES					
Satisfaction with Marital Interaction	1.65	0.55	1.56–1.73	1.00–3.00	0.894
Satisfaction with Emotional Aspects ^d^	1.97	0.56	1.88–2.05	1.00–3.00	0.789
Satisfaction with Structural Aspects ^e^	1.70	0.50	1.63–1.78	1.00–3.00	0.829

^a^ World Health Organization Quality of Life, abbreviated version; ^b^ Marital Satisfaction Scale; ^c^ 95% Confidence Interval; ^d^ Satisfaction with partner’s emotional aspects; ^e^ Satisfaction with structural aspects, organization way, establishment and compliance of rules by the partner.

**Table 3:** Factors associated with scores of WHOQOL-BREF ^a^ domains of married family caregivers of patients with mental disorders, multiple linear regression.

FACTORS	Physical Health Domain R^2^ = 0.156			Psychological Domain R^2^ = 0.239			Social Relationships Domain R^2^ = 0.044			Environment Domain R^2^ = 0.135		
	B	95% CI	*p*-Value	B	95% CI	*p*-Value	B	95% CI	*p*-Value	B	95% CI	*p*-Value
RELATED TO CAREGIVER												
Male gender	9.40	4.43; 14.38	0.000	7.79	4.03; 11.55	0.000	4.68	−1.03; 10.39	0.108	4.00	−1.77; 9.78	0.173
Age in years	−0.10	−0.34; 0.15	0.438	−0.19	−0.37; −0.001	0.048	−0.21	−0.49; 0.07	0.145	0.10	−0.19; 0.38	0.498
≤8 years of schooling	−0.68	−6.18; 4.81	0.807	−1.39	−5.54; 2.76	0.509	1.54	−4.76; 7.85	0.630	−6.69	−13.07; −0.31	0.040
≥5 years as caregiver	5.90	0.80; 11.00	0.024	4.51	0.66; 8.36	0.022	3.52	−2.34; 9.37	0.237	6.59	0.67; 12.51	0.029
Support by relatives	−1.00	−6.02; 4.02	0.695	2.00	−1.80; 5.79	0.300	−2.19	−7.95; 3.58	0.454	−0.34	−6.18; 5.49	0.907
RELATED TO PATIENT												
Male gender	−2.65	−7.83; 2.53	0.314	−0.32	−4.23; 3.60	0.873	−0.49	−6.44; 5.45	0.870	−1.79	−7.80; 4.22	0.558
Age in years	−0.09	−0.30; 0.12	0.394	−0.02	−0.17; 0.14	0.827	0.01	−0.23; 0.25	0.929	0.10	−0.14; 0.34	0.408
Crisis in the last 30 days	−7.00	−12.81; −1.18	0,019	−9.49	−13.88; −5.09	0.000	−4.88	−11.55; 1.79	0.151	−8.55	−15.30; −1.80	0.013

^a^ World Health Organization Quality of Life, abbreviated version.

**Table 4:** Factors associated with MSS ^a^ subscales of family caregivers of patients with mental disorders, multiple linear regression.

VARIABLES	Satisfaction with Marital Interaction R^2^ = 0.100			Satisfaction with Emotional Aspects R^2^ = 0.177			Satisfaction with Structural Aspects R^2^ = 0.129		
	B	95% CI	*p*-Value	B	95% CI	*p*-Value	B	95% CI	*p*-Value
RELATED TO CAREGIVER									
Male gender	−0.17	−0.34; 0.00	0.050	−0.18	−0.35; −0.01	0.033	−0.24	−0.39; −0.09	0.002
Age in years	−0.002	−0.01; 0.01	0.609	−0.0001	−0.01; 0.01	0,985	0.002	−0.01; 0.01	0.554
≤8 years of schooling	0.22	0.03; 0.41	0.025	0.15	−0.03; 0.34	0.107	0.10	−0.07; 0.27	0.253
≥ 5 years as caregiver	−0.06	−0.23; 0.12	0.521	−0.16	−0.33; 0.01	0.064	0.03	−0.13; 0.19	0.716
Support by relatives	−0.18	−0.35; −0.01	0.044	−0.27	−0.44; −0.10	0.002	−0.24	−0.39; −0.09	0.002
RELATED TO PATIENT									
Male gender	−0.04	−0.22; 0.14	0.666	−0.05	−0.22; 0.13	0.583	−0.05	−0.21; 0.10	0.500
Age in years	0.002	−0.01; 0.01	0.553	−0.004	−0.01; 0.003	0.306	0.001	−0.01; 0.01	0.717
Crisis in the last 30 days	0.10	−0.10; 0.30	0.342	0.21	0.01; 0.40	0.040	0.03	−0.15; 0.21	0.741

^a^ Marital Satisfaction Scale.
